# Rhizosphere and soil metagenomes and metagenome-assembled genomes from the Byers Peninsula, Livingston Island (62°S), Antarctica

**DOI:** 10.1128/mra.00171-25

**Published:** 2025-06-09

**Authors:** Valentín Berríos-Farías, Sergio Guajardo-Leiva, Jorge Gallardo-Cerda, Cristóbal Galbán-Malagón, Gabriel Ballesteros, Claudia Egas, Marco Molina-Montenegro, Eduardo Castro-Nallar

**Affiliations:** 1 Departamento de Microbiología, Facultad de Ciencias de la Salud, Universidad de Talca, Campus Talca, Talca, Chile; 2 Centro de Ecología Integrativa, Universidad de Talca, Campus Talca, Talca, Chile; 3 Instituto de Ciencias Biológicas, Universidad de Talca, Campus Talca, Talca, Chile; 4 Centro de Genómica, Ecología y Medio Ambiente (GEMA), Universidad Mayor, Santiago, Chile; 5 Institute of Environment, Florida International University 5450, Miami, Florida, USA; 6 Data Observatory Foundation, Santiago, Chile; 7 Centro de Investigación en Estudios Avanzados del Maule (CIEAM), Universidad Católica del Maule 28048, Talca, Chile; Montana State University, Bozeman, Montana, USA

**Keywords:** desert microbiome, Antarctic vascular plants, shotgun metagenomics

## Abstract

Rhizosphere microbes establish functional interactions with their hosts, impacting plant fitness. To further understand plant effects on microbial composition and functional diversity, we present 52 metagenomes and 1,484 metagenome-assembled genomes (MAGs) from soil and the rhizosphere of *Colobanthus quitensis* and *Deschampsia antarctica*.

## ANNOUNCEMENT

Plants form intimate and functional relationships with microbes that can be mutually beneficial (1). However, it is essential to study how plant species specifically recruit microbes (2, 3). For instance, rhizosphere microbial communities may assemble from the soil species pool through chemical signals from the plant, which select specific phylogenetic lineages. Conversely, plant effects might favor bacterial functions over lineages, partly explaining the observed heterogeneity in rhizosphere compositions (4, 5).

We collected naked soil and root samples from Antarctic vascular plants, *Colobanthus quitensis* and *Deschampsia antarctica,* located in the Byers Peninsula, Livingston Island, Antarctica (62°S; sample data in https://figshare.com/s/89fd6b3b3548dc4610a9?file=54438623). We obtained rhizosphere-associated soil (RSS), which is soil not in contact with roots but part of the root mesh, and rhizosphere, which is soil in physical contact with roots, from root samples. Samples were processed under a biosafety cabinet (BSC-class II), where a 250 mg aliquot was taken with a sterile spatula for DNA extraction using Qiagen’s QIAmp PowerFecal DNA extraction kit. The total amount of DNA extracted per sample is reported in https://figshare.com/s/89fd6b3b3548dc4610a9?file=54438623. Paired-end sequencing libraries were constructed using Illumina TruSeq DNA kits and sequenced on an Illumina NovaSeq 6000, producing 3,364,573,092 raw reads of 150 bp.

**Fig 1:**
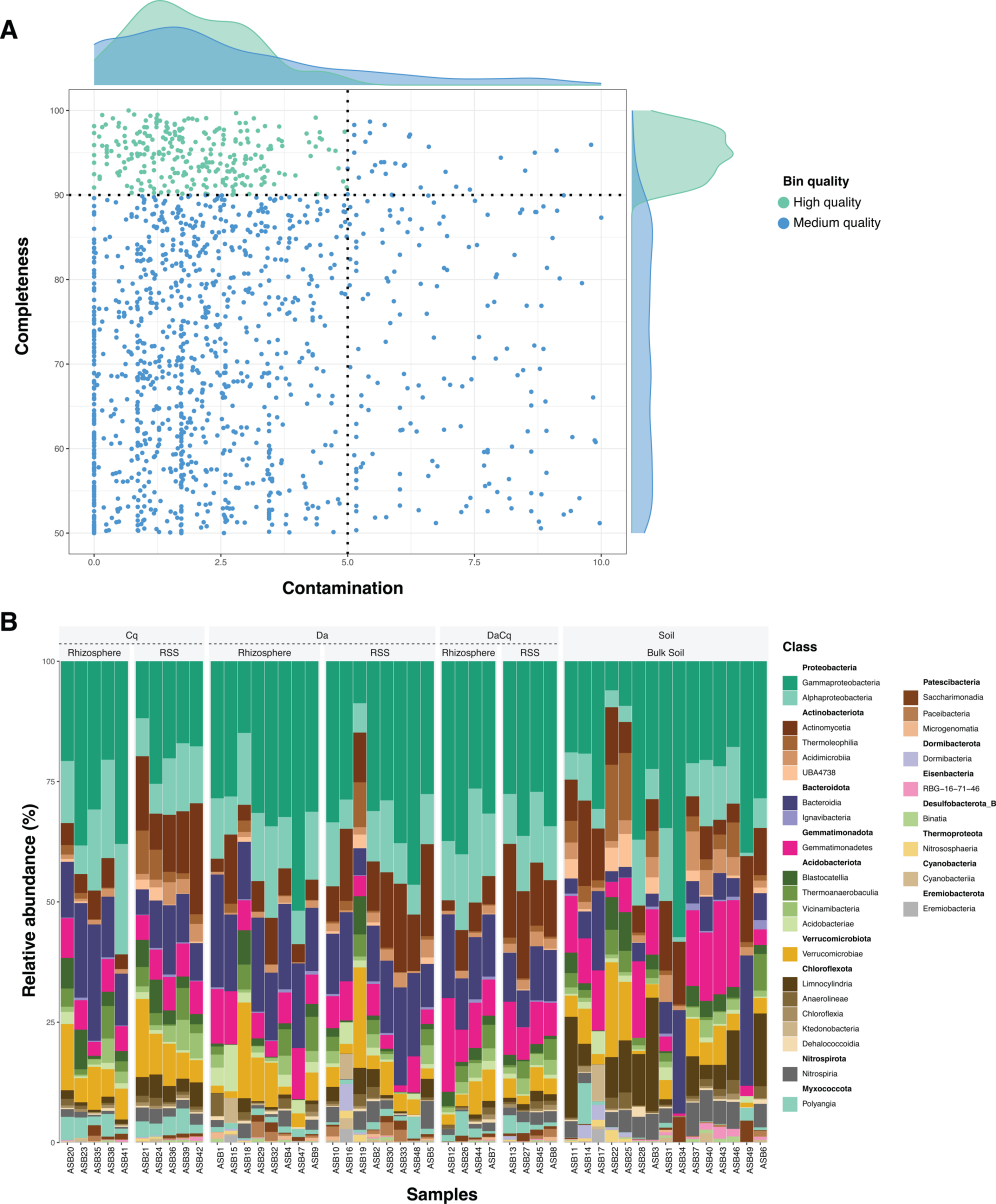
Bacterial community composition in Byers Peninsula soil and rhizosphere represented by MAGs. (A) A scatter plot displays completeness and contamination metrics for inferred MAGs, estimated using CheckM. Cyan dots indicate high-quality MAGs (completeness >90%, contamination <5%), while blue dots denote medium-quality MAGs (completeness ≥ 50%, contamination <10%). Dotted lines on the X and Y axes show thresholds for contamination and completeness. Density plots illustrate the quantity of MAGs in each scatterplot region, attached to both axes. (B) A stacked bar plot shows the relative abundances of bacterial classes. These abundances are arranged in grids by sample type: bulk soil, rhizospheres, and rhizosphere surrounding soils (RSS) from *Colobanthus quitensis* (Cq) and *Deschampsia antarctica* (Da). Additionally, samples from the combined Da and Cq rhizospheres are included (DaCq).

We employed the default software settings unless stated otherwise. We removed adapters (--detect_adapter_for_pe) and filtered and trimmed reads (-q 30 L 100) using fastp version 0.21.0 (6), which resulted in 2,528,781,138 filtered reads. We first estimated MASH distances and checked whether metagenomes form clusters using the Hopkins statistics (> 0.75) (7). We then used agglomerative hierarchical clustering (hclust function in the base R package), which resulted in four clusters, as suggested by silhouette analysis. We then performed reads co-assembly for each metagenomic cluster (https://figshare.com/s/89fd6b3b3548dc4610a9?file=54438686). We used MEGAHIT version 1.2.9 for metagenomic clusters 1 and 2 and SPAdes version 3.15.5 for clusters 3 and 4 (8, 9). To bin the resulting contigs, we used VAMB version 3.0.2, CONCOCT version 1.1.0, and MetaBAT2 version 2.15 for each of the co-assembly clusters before refining the resulting bins using the bin refinement module implemented in MetaWRAP version 1.3.2. (-c 50 x 10) (10–13). The set of 1,484 MAGs was assessed using CheckM version 1.2.0, yielding 292 high-quality and 1,192 medium- and low-quality MAGs based on conserved marker genes (14, 15) (Fig. 1A; https://figshare.com/s/89fd6b3b3548dc4610a9?file=54438620). Bins were dereplicated at a 99% ANI threshold, and representative bins were chosen based on CheckM genome quality values. Finally, we calculated the contigs’ mean coverage per bin using CoverM version 0.6.1 to estimate MAG abundances (--min-read-aligned-percent 30 –m mean --dereplicate --dereplication-ani 99 --dereplication-precluster-method finch) (https://github.com/wwood/CoverM). Bins were previously dereplicated with a 99% ANI threshold, and the representative bin was chosen based on CheckM genome quality values.

At the phylum level, communities were dominated by Proteobacteria (38.8%) and Actinobacteriota (14.8%), followed by Bacteroidota (12%), Gemmatimonadota (7.8%), Acidobacteriota (7.8%), Verrucomicrobiota (5.9%), and Chloroflexota (5.6%). Likewise, at the family level, the most abundant MAGs belonged to Burkholderiaceae (11.9%), Chitinophagaceae (8.4%), Sphingomonadaceae (7.3%), Gemmatimonadaceae (5.6%), Rhodanobacteraceae (4.6%), UBA10450 (4.4%), and Dermatophilaceae (4.2%) (Fig. 1B).

## Data Availability

The entire metagenome shotgun project is archived in GenBank with BioProject accession number PRJNA746701. The version presented in this paper is the initial version.
